# Tracheal Capillary Hemangioma Successfully Treated With Combined Bronchoscopic Cryotherapy and Argon-Plasma Coagulation

**DOI:** 10.7759/cureus.18547

**Published:** 2021-10-06

**Authors:** Parinya Ruenwilai, Chalerm Liwsrisakun, Atikun Limsukon, Chaiwat Bumroongkit, Nirush Lertprasertsuk

**Affiliations:** 1 Division of Pulmonary, Critical Care and Allergy, Department of Internal Medicine, Faculty of Medicine, Chiang Mai University, Chiang Mai, THA; 2 Department of Pathology, Faculty of Medicine, Chiang Mai University, Chiang Mai, THA

**Keywords:** argon plasma coagulation, cryotherapy, bronchoscopy, tracheal lobular capillary hemangioma, primary tracheal tumor

## Abstract

We report a 43-year-old female who presented with recurrent non-massive hemoptysis for four months. Chest radiograph was normal. Thoracic computed tomography (CT) scan revealed a 0.8-centimeter intraluminal polypoid mass abutting the middle part of trachea. A fiber-optic bronchoscopy demonstrated a lobulated reddish mass at dorsolateral aspect of the right side of tracheal wall. Cryotherapy to remove the mass and argon plasma coagulation to stop bleeding at the stump was performed. The histological evaluation conﬁrmed the diagnosis of a tracheal lobular capillary hemangioma. There was no recurrence hemoptysis nor the recurrence of tumor during a one-year follow-up.

## Introduction

Primary tumors of the trachea are uncommon and they are mostly malignant in nature [1]. Tracheal lobular capillary hemangioma (LCH) is an extremely rare benign primary tumor of the trachea, particularly in adults [2]. Here, we report a previously healthy female presenting with non-massive hemoptysis caused by tracheal LCH.

## Case presentation

**Figure 1:**
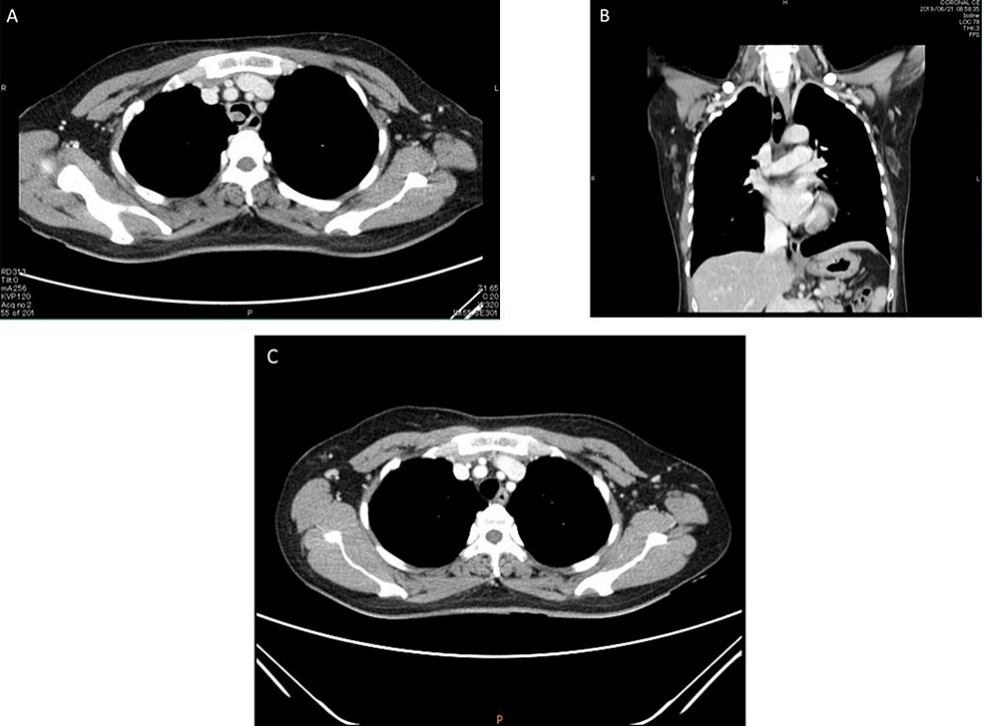
A and B: Computerized tomography chest scan, demonstrating a 0.8-cm polypoid lesion abutting right side of the middle part of the trachea, C: Computerized tomography chest scan performed 8 months later showed no evidence of tumor recurrence

**Figure 2:**
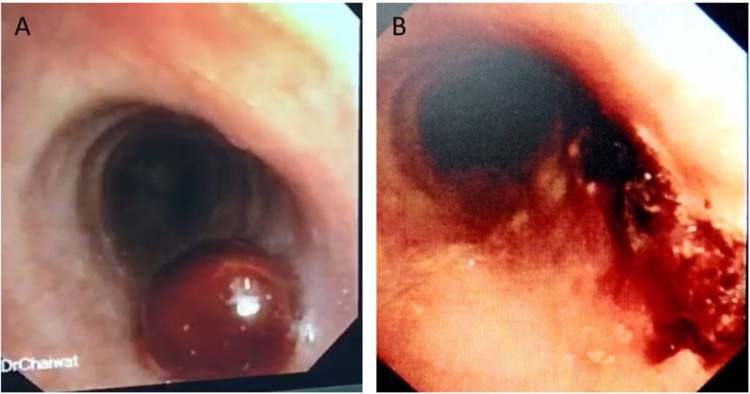
A: Bronchoscopy showed a 0.8-cm. lobulated cherry-red mass at the right side of the tracheal wall, B: Minimal postoperative bleeding which responded well to argon plasma coagulation

**Figure 3:**
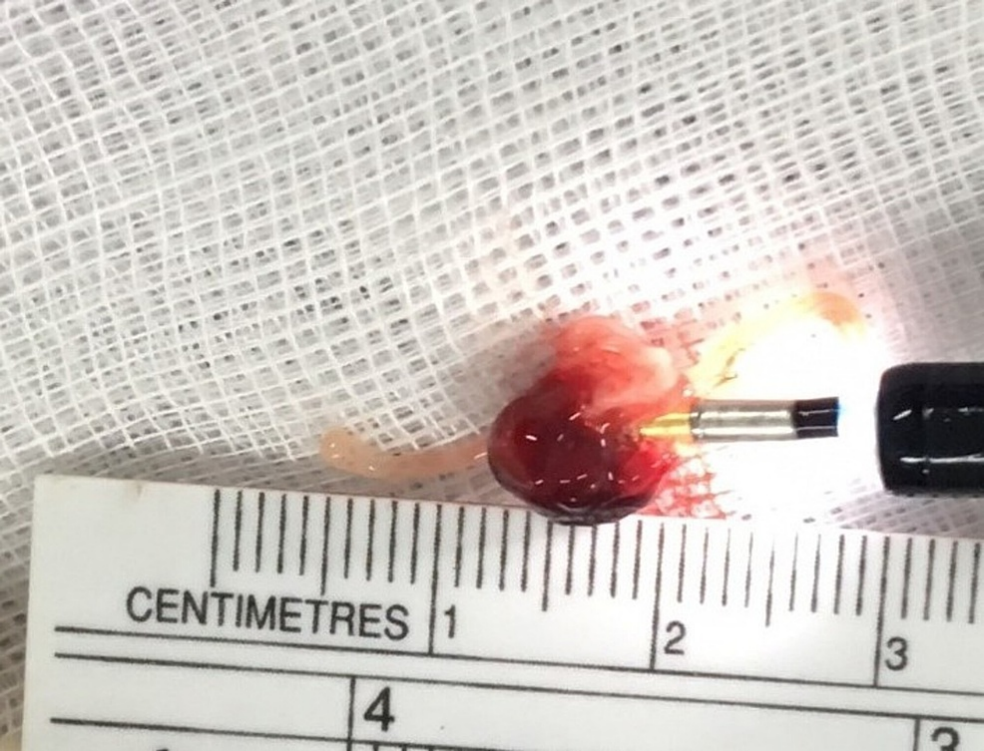
Tracheal hemangioma mass with stalk removed by cryotherapy

**Figure 4:**
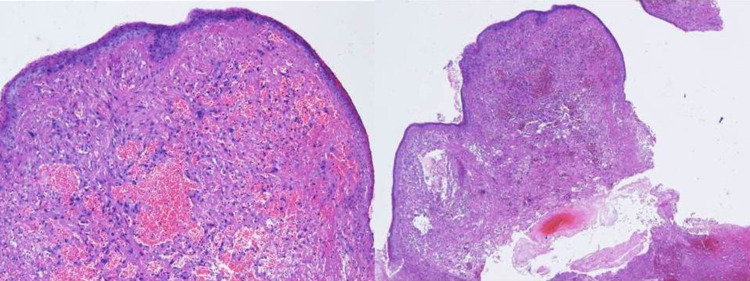
Microscopic examination showed organized stroma with hyaline deposits, entrapped red blood cells in the slits among the vascular channels underneath the erosive mucosal surface with granulation tissue, and organized hematoma

**Figure 5:**
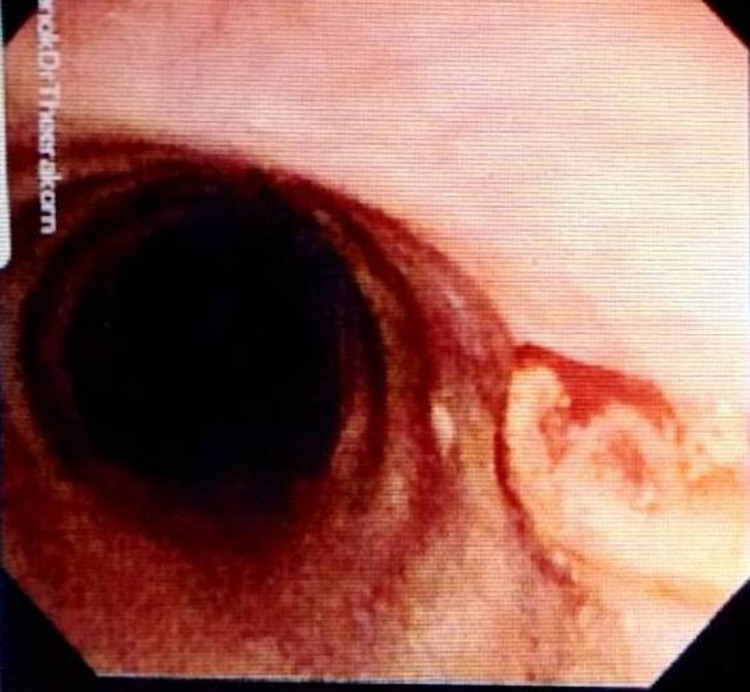
Follow-up bronchoscopy demonstrated healing of excision site without evidence of tumor recurrence

A 43-year-old non-smoking female, previously healthy, came to the hospital with history of chronic cough and blood-streaked sputum for four months. She had neither symptoms of wheezing nor dyspnea. Four weeks before hospitalization, she developed difficulty breathing during inspiration. Chest auscultation demonstrated inspiratory stridor over the upper chest. Her chest radiograph was normal, however the thoracic computed tomography (CT) scan for initial evaluation of hemoptysis revealed a 0.8-cm intraluminal polypoid mass with tumor contrast enhancement of 43.1 HU abutting the right side of the middle part of trachea (Figure 1A, 1B). Fiber-optic bronchoscopy, performed under light sedation with intravenous midazolam, showed a 0.8-cm lobulated cherry-red mass, suggestive of vascular tumor, at the right side of the tracheal wall (Figure 2A). It was located 5.5 cm distal to the vocal cords. We decided to performed cryotherapy without initial forceps biopsy for both diagnostic and therapeutic purposes. Pre-intervention tracheal intubation through fiberoptic bronchoscope, without general anesthesia, to secure the airway in case of bleeding was instituted. Cryorecanalization to remove the entire mass (Figure 3) was successfully done with minimal postoperative bleeding which responded well to argon plasma coagulation (Figure 2B). On pathologically microscopic examination, organized stroma with hyaline deposits, entrapped red blood cells in the slits among the vascular channels were observed beneath the erosive mucosal surface with granulation tissue and organized hematoma (Figure 4). These findings were compatible with the diagnosis of a tracheal capillary hemangioma. After bronchoscopic intervention, the patient was discharged without any complications the following day. Three months later, follow-up bronchoscopy demonstrated healing of the excision site without evidence of tumor recurrence (Figure 5). There was no recurrent hemoptysis during one-year follow-up. Chest CT performed eight months later showed no evidence of tumor recurrence (Figure 1C).

## Discussion

Adult tracheal LCH is very rare, with fewer than 20 cases reported in the literature so far [1,3]. Tracheal LCH can arise spontaneously or secondary from trauma for example post-tracheal intubation or after airway foreign body aspiration [4]. The pathogenesis is not clear. Imbalance of sex hormones during pregnancy, viral and bacterial infections, microscopic arteriovenous malformations, disequilibrium of vascular growth factors, and cytogenetic abnormalities are believed to be the etiology [1].

The most common clinical presentations of tracheal LCH are recurrent cough and hemoptysis due to hypervascularization of the tumor [1]. Hemoptysis is usually non-massive as our patient. However, it can present as massive tracheobronchial bleeding [5]. Symptoms of airway obstruction are uncommon, however, a large tracheal LCH can present as life-threatening severe refractory asthma [6].

Chest X-ray findings in tracheal LCH, like our patient, are generally normal. Thoracic CT is more sensitive than standard chest radiography for the diagnosis of endotracheal lesion. Although CT findings are usually non-specific, a densely homogeneous contrast enhancement with an average density of more than 100 HU is very suggestive of tracheal LCH [2]. Tracheal adenoma, the more common benign tracheal tumor, may be significantly contrast-enhancing but it will be in the slower rate of enhancement in comparison with LCH [2]. Popcorn calcification, like hamartoma, has been reported in LCH [7]. However, fat tissue will not be dominant within the lesion as the characteristic of hamartoma [2].

Bronchoscopic appearance is typically glittering sessile or polypoid cherry-like lesion [8]. This classic bronchoscopic finding, like our case, is characteristic of a new lesion. The long-standing lesion may present as collagenous nodule indistinguishable from other tumors [1]. Tracheal LCH usually presents as a single lesion and uncommonly multiple nodules [2]. The size is usually smaller than 10 mm. The biggest one ever reported was 2x4 cm, which caused airflow obstruction in a pregnant woman [6]. Definitive diagnosis is made by bronchoscopic biopsy. Because of its highly vascularized property, the risk of bleeding from diagnostic biopsy or tumor removal by intervention is high [4]. In our case, the endoscopic finding of tumor made us change the plan from forceps biopsy to cryotherapy instead. Histological evaluation tracheal LCH showed various sizes of capillaries arranged in lobules which were partitioned by edematous collagenous and fibrous stroma [1,9].

The benefit of bronchoscopy is not for only diagnosis, but also definite treatment. Interventional bronchoscopy has been reported to successfully treat tracheal LCH. Various methods, including Nd-YAG laser therapy, electrocautery, excision by biopsy forceps, brachytherapy or cryotherapy through either rigid or fiberoptic bronchoscope, have been used for treatment of this tumor [1,3]. In our case we preferred using cryoprobe for removal of tumor via flexible fiber-optic bronchoscopy under endotracheal tube because it is safer to secure the airway in case of bleeding, less expensive, and no need for general anesthesia and operating room. Benefits of cryotherapy include its freezing property to stop bleeding in this highly vascularized tumor. In addition, it has a lower risk of immediate complications such as perforation and late complication of tracheal stenosis or tracheomalacia as the result of cryoresistant property of tracheal cartilaginous rings [10]. In our case, after tumor removal with cryotherapy, minimal stump bleeding was stopped using argon plasma coagulation (APC). Our technique using cryotherapy in combination with APC for treatment of tracheal LCH was the same as reported by Chen et al. [9]. Outcome of treatment was good without any complication. No bronchoscopic and CT imaging evidences of tumor recurrence were observed for eight weeks and eight months after the procedure, respectively. The patient was free of symptoms for up to one year after the intervention.

## Conclusions

Although tracheal CH is a rare cause of radiologically-negative hemoptysis, it should be kept in mind for differential diagnosis. Thoracic CT is very sensitive for identifying tracheal tumor and diagnosis of tracheal LCH should be suspected in case of densely homogeneous contrast-enhancement of the lesion. Bronchoscopy is beneficial for both definite diagnosis and treatment. Interventional bronchoscopy using combined cryotherapy and argon plasma coagulation demonstrates excellent outcomes without any significant acute and long-term complications.
